# Circulating Human Metabolites Resulting from TOTUM-070 Absorption (a Plant-Based, Polyphenol-Rich Ingredient) Improve Lipid Metabolism in Human Hepatocytes: Lessons from an Original Ex Vivo Clinical Trial

**DOI:** 10.3390/nu15081903

**Published:** 2023-04-14

**Authors:** Fabien Wauquier, Line Boutin-Wittrant, Stéphanie Krisa, Josep Valls, Cedric Langhi, Yolanda F. Otero, Pascal Sirvent, Sébastien Peltier, Maxime Bargetto, Murielle Cazaubiel, Véronique Sapone, Annie Bouchard-Mercier, Véronique Roux, Nicolas Macian, Gisèle Pickering, Yohann Wittrant

**Affiliations:** 1 Clinic’n’Cell SAS, Faculty of Medicine and Pharmacy, 63001 Clermont-Ferrand, France; fabien_wauquier@gmx.fr (F.W.); linewittrant@gmail.com (L.B.-W.); 2 Institut des Sciences de la Vigne et du Vin, Bordeaux INP, INRAE, OENO, UMR 1366, University of Bordeaux, 33140 Villenave d’Ornon, France; stephanie.krisa@u-bordeaux.fr (S.K.); josep.valls-fonayet@u-bordeaux.fr (J.V.); 3 Bordeaux Metabolome, MetaboHUB, 33140 Villenave d’Ornon, France; 4 Valbiotis, R&D Riom Center, 63200 Riom, France; cedric.langhi@valbiotis.com (C.L.); yolanda.otero@valbiotis.com (Y.F.O.); pascal.sirvent@valbiotis.com (P.S.); 5 Valbiotis, R&D Périgny Center, 17180 Périgny, France; sebastien.peltier@valbiotis.com (S.P.); bargetto.maxime@gmail.com (M.B.); murielle.cazaubiel@valbiotis.com (M.C.); veronique.sapone@valbiotis.com (V.S.); 6 Valbiotis, R&D Quebec Center, Québec, QC G1V 0A6, Canada; annie.bouchard@valbiotis.com; 7 CIC INSERM 1405, Plateforme d’Investigation Clinique CHU Gabriel Montpied, 63000 Clermont-Ferrand, France; v_morel@chu-clermontferrand.fr (V.R.); nmacian@chu-clermontferrand.fr (N.M.); gisele.pickering@uca.fr (G.P.); 8 INRAE, UMR 1019, UNH, 63009 Clermont-Ferrand, France; 9 Faculty of Medicine and Pharmacy, Clermont Auvergne University, UMR1019 of Human Nutrition, 63000 Clermont-Ferrand, France

**Keywords:** clinical trial, ex vivo, lipid metabolism, cholesterol, hepatocytes

## Abstract

TOTUM-070 is a patented polyphenol-rich blend of five different plant extracts showing separately a latent effect on lipid metabolism and potential synergistic properties. In this study, we investigated the health benefit of such a formula. Using a preclinical model of high fat diet, TOTUM-070 (3 g/kg of body weight) limited the HFD-induced hyperlipemia with a reduction in triglyceride (−32% after 6 weeks; −20.3% after 12 weeks) and non-HDL cholesterol levels (−21% after 6 weeks; −38.4% after 12 weeks). To further investigate such a benefit and its underlying mechanisms in humans, we designed an ex vivo clinical approach to collect the circulating bioactives resulting from TOTUM-070 ingestion and to determine their biological activities on human hepatocytes. Human serum was obtained from healthy subjects before and after intake of TOTUM-070 (4995 mg). The presence of circulating metabolites was assessed by UPLC-MS/MS. Serum containing metabolites was further incubated with hepatocytes cultured in a lipotoxic environment (palmitate, 250 µM). RNA sequencing analyses show that lipid metabolism was one of the most impacted processes. Using histologic, proteomic, and enzymatic assays, the effects of human TOTUM-070 bioactives on hepatocyte metabolism were characterized by (1) the inhibition of lipid storage, including both (2) triglycerides (−41%, *p* < 0.001) and (3) cholesterol (−50%, *p* < 0.001) intracellular content, (4) a reduced de novo cholesterol synthesis (HMG-CoA reductase activity −44%, *p* < 0.001), and (5) a lowered fatty acid synthase protein level (*p* < 0.001). Altogether, these data support the beneficial impact of TOTUM-070 on lipid metabolism and provide new biochemical insights in human mechanisms occurring in liver cells.

## 1. Introduction

Hypercholesterolemia is a disorder of lipid metabolism, which corresponds to an increase in cholesterol levels in the blood. Hypercholesterolemia progressively affects the arterial walls [1] promoting local inflammation and driving the recruitment of foamy macrophages that infiltrate to form the atherosclerotic plaque [2]. Thus, prolonged hypercholesterolemia drives atherosclerosis onset and therefore contributes to severe cardiovascular (CV) system dysfunctions including ischemic heart disease (IHD). To date, IHD is the world’s first cause of death representing 84.5% of cardiovascular deaths and about 28.2% of all-cause mortality [3,4].

To address such a health condition, strategies targeting improved cholesterol metabolism to prevent cardiovascular issues have become a quest. Statins have a well acknowledged role as a first-line therapeutic option for lowering LDL-c with demonstrated efficacy on CV risk reduction [5]. However, in some cases, patients may be unable to tolerate statin doses that would be necessary to efficacy lower LDL-c [6]. This intolerance mostly relies on muscle-related side effects [7,8]. In this light, alternative strategies such as nutritional preventive approaches may represent a safer option of major interest especially for individuals presenting a higher risk of CVD associated or not either with a modest hypercholesterolemia or hypertriglyceridemia.

Nutrition is a major environmental factor capable of impacting significantly and lastingly several biological functions. To date, functional foods such as low-fat spread enriched with plant sterols and/or fish omega-3 fatty acids were reported to lower serum triglyceride and LDL-cholesterol concentrations in individuals with modest hypercholesterolemia and hypertriglyceridemia [9]. Bergamot phytosomes improve plasma lipid profiles in overweight and obese people with mild hypercholesterolemia [10]. Oat fibers have demonstrated prebiotic abilities leading to a modulation of gut microbia associated with increased short-chain fatty acids and lowered total cholesterol and non-high-density lipoprotein cholesterol (non-HDL-C) in plasma [11].

Among other nutrients of interest, polyphenols are widely studied in the scientific literature. These compounds participate to the immune defense mechanisms of plants. From a chemical point of view, these organic molecules are characterized by the presence of one or more phenolic groups. Biologically, polyphenols are known for their antioxidant and anti-inflammatory properties [12]. This health benefit is particularly described for the cardiovascular system [13]. However, clinically validated approaches and further investigations on the mode of action at cellular level especially in humans are required for optimized care management.

TOTUM-070 is a patented polyphenol-rich blend of five different plant extracts showing separately a latent effect on lipid metabolism and potential synergistic properties. The objective of this study was to demonstrate whether and how this combination of different polyphenols can improve lipid metabolism in order to support health care management in the mid to moderate hypercholesterolemia population. In the first part of this manuscript, we demonstrated and validated such a benefit in a preclinical model of a high-fat diet. Then, using an ex vivo clinical approach that considers the whole digestive process of nutrients [14,15,16,17,18], we investigated the potential health benefit of human circulating bioactive metabolites resulting from TOTUM-070 ingestion.

In this quest to improve lipid metabolism, the hepatocyte stands as the cellular armed wing [16,19], and the regulation of cholesterol metabolism pathways in hepatocytes represents the most relevant means [20,21]. Therefore, we examined whether and how human metabolites derived from TOTUM-070 ingestion may regulate the activities of hepatocytes to influence lipid metabolism in order to support preventive strategies in hypercholesterolemia and subsequent atherosclerosis management.

## 2. Materials and Methods

### 2.1. Study Product

VALBIOTIS has developed TOTUM-070 (priority French patent: FR1559965), a food ingredient composed by the combination of different plant extracts. The extracts were initially selected on the basis of published clinical data and/or traditional use for their possible effectiveness in reducing LDL-c.

### 2.2. Ethics Preclincal Study

Animal procedures were reviewed by the local ethics committee (C2E2A, Auvergne, France). Male Syrian golden hamsters aged 6 weeks were used for this study (Janvier Labs, Le Genest-Saint-Isle, France).

### 2.3. Ethics Clinical Trial

The study was conducted in accordance with the Declaration of Helsinki of 1975 (https://www.wma.net/what-we-do/medical-ethics/declaration-of-helsinki (accessed on 20 May 2021)) revised in 2013. The human study was approved by the French Ethical Committee (2021-071 RIPH2 HPS/N° SI RIPH: 21.05.18.40345/N° EudraCT/ID RCB: 2021-A01211-40/Comité de Protection des Personnes CPP Sud-Est I; approved 18 June 2021). The volunteers were informed of the objectives and the potential risks of the present study and provided their written informed consent before they participated in the study.

### 2.4. Preclincal Protocol

Six-week-old Syrian golden hamsters were received and housed at the Valbiotis animal facility (Valbiotis, Riom, France) under standard 12 h light and 12 h dark cycle. Upon arrival, all hamsters were fed a low-fat diet (LFD, D18102403, Research Diet, New Brunswick, NJ, USA) for 2 weeks of acclimatization until the age of 8 weeks. Thereafter, hamsters were placed either on a high fat high cholesterol diet (HFD: 45 kcal% fat mainly hydrogenated coconut oil, 17 kcal% fructose, 1.4 gm% cholesterol, D99122211, Research Diet, New Brunswick, NJ, USA) or maintained on a LFD. The hamsters placed on HFD were divided in two groups: a control group (*n* = 22) and a treated group supplemented with TOTUM-070 (*n* = 20). TOTUM-070 solution was prepared in vehicle tap water Tween20 1% at 300 g/L. Administration of TOTUM-070 in HFD hamsters was performed by daily gavages at 3 g/kg body weight. The HFD control group and the LFD group were gavaged with vehicle only. Food and water were supplied ad libitum. Study duration was completed after 12 weeks of supplementation. Lipid parameters were monitored in the blood before the study started (Study Week 0, SW0) and after 6 weeks of supplementation (SW6) at the end of the study (SW12).

### 2.5. Lipid Parameters in Preclinical Study

The hamsters were fasted for 6 h, and blood was collected from the gingival vein. After collection, blood was incubated at room temperature for 30 min then centrifuged for 10 min at 2000× *g*. Serum was harvested and stored at −80 °C until ready for analysis. HDL-enriched serum fractions were isolated after treatment of serum samples with LDL/VLDL precipitation buffer (ref ab105138, Abcam, Boston, MA, USA). The cholesterol levels in the serum (total cholesterol) and in the purified HDL fractions (HDL-cholesterol) were quantified enzymatically using a CHOD-PAP colorimetric assay kit (Ref 80106, Biolabo SAS, Maizy, France). The non-HDL-cholesterol fraction was calculated by subtraction the HDL-cholesterol concentration from total cholesterol concentration for each sample. The serum triglyceride levels were measured using a colorimetric assay kit (Ref 10010303, Cayman chemical, Ann Arbor, MI, USA).

### 2.6. Human Study Design and Pharmacokinetic of Absorption

A total of 10 healthy men (age: 24.6 years old, ±5.2; BMI: 22.55 kg/m^2^, ±1.7; >60 kg; without any pathologies nor drug treatment; and no distinction in ethnicity) volunteered for this study. They were validated for normal blood formulation, renal (urea and creatinine), and liver functions. Parameters included aspartate aminotransferase (AST), alanine aminotransferase (ALT), and gamma-glutamyltransferase (GGT) activities. Blood samples from all participants were collected in serum-dedicated tubes.

The first clinical phase of the project was aimed at determining TOTUM-070’s metabolites absorption peak. Ten healthy volunteers were fasted for 12 h prior to be given 4995 mg of TOTUM-070. TOTUM-070 was given as eight capsules. Approximately 9 mL of venous blood were collected before the ingestion and every 20 min for 240 min after the ingestion of TOTUM-070. Blood samples were obtained from the median cubital vein. Serum was collected, aliquoted, and stored until analyses at −80 °C at the Centre d’Investigation Clinique de Clermont-Ferrand—Inserm 1405. This dedicated research department is fully compliant with regulatory and ethical clinical obligations (certification according to the French standard NF S 96900).

Then, human circulating metabolites from polyphenols were quantified and characterized by ultra-high performance liquid chromatography hyphenated with tandem mass spectrometry (UPLC-MS/MS). Once the absorption profile was characterized, volunteers were asked to come one more time to the clinical center for the collection of the metabolites’ enriched serum fraction. For this second clinical phase, ten healthy volunteers fasted for 12 h prior to being given 4995 mg of TOTUM-070. Approximately 48 mL of venous blood were collected before the ingestion for the collection of a naïve serum (baseline). Then, after the ingestion, at Cmax (absorption profil’s peak), 48 mL of blood were collected for circulating bioactives collection. Blood samples were obtained from the median cubital vein. The serum was stored at −80 °C until required for analysis.

### 2.7. Phenolic Compounds Extraction from Serum

Serum (0.9 mL) was mixed with 2.7 mL of methanol 100% for 1 min. The resulting mixture was centrifuged at 20,000× *g* for 15 min. The supernatant was collected in separated tubes and then evaporated to dryness using a SpeedVac Concentrator (Thermo Fisher Scientific, Illkirch, France). The dried material was dissolved in 80 µL of methanol/water (50:50 *v*/*v*). After agitation (1 min), ultrasonication (1 min), and centrifugation (20,000× *g* for 15 min), the supernatant was stored at −20 °C until ready for analysis by ultra-high performance liquid chromatography hyphenated with tandem mass spectrometry.

### 2.8. Ultra-High Performance Liquid Chromatography-Mass Spectrometry (UPLC-MS/MS)

The analyses of phenolic compounds were carried out using a 1260 Infinity UHPLC (Agilent Technologies). The UHPLC system was coupled to a 6430 triple quadrupole mass spectrometer from Agilent Technologies. Four µL were injected into a Zorbax SB-C_18_ column (2.1 × 100 mm, 1.8 µm) (Agilent Technologies, Les Ulis, France). Two different solvents were used as a mobile phase: solvent A (water/formic acid 99.9:0.1, *v*/*v*) and solvent B (acetonitrile/formic acid 99.9:0.1, *v*/*v*), at a flow rate of 0.3 mL/min and a gradient as follows in solvent A: 0 min 1% B, 2 min 5% B, 3 min 25% B, 6 min 25% B, 8 min 40% B, 11.5 min 95% B, 14 min 95% B, and 16 min 1% B. The MS/MS parameters were set as follows: negative ion mode, capillary tension 3000 V, nebulizer 15 psi, dry gas 11 L/min, dry temperature 350 °C, and acquisition in multiple reaction monitoring (MRM), see Supplementary Table S1 for further details. The data were processed using MassHunter software (Agilent Technologies).

### 2.9. Human Hepatocyte Cultures

The human hepatocyte cell line HepG2 was obtained from the European Collection of Authenticated Cell Cultures and purchased from Sigma-Aldrich (Saint-Quentin-Fallavier, France, 85011430). During the maintenance phase, HepG2 cells were cultured in Dulbecco’s modified Eagle’s medium (Invitrogen, Carlsbad, CA, USA) with 10% fetal bovine serum (Invitrogen) and 1% penicillin/streptomycin (Life Technologies, Villebon-Sur-Yvette, France). All cell cultures were performed at 37 °C in an atmosphere of 5% CO_2_/95% air. To analyze the effects of TOTUM-070 metabolites, the cells were preincubated for 24 h in DMEM in the presence of 10% of human serum (naïve or containing circulating metabolites) according to the Clinic’n’Cell methodology (DIRV INRAE 18-0058) prior to an additional 48 h incubation in a palmitate-induced lipidic stress environment (palmitate 250 µM; Sigma, Saint-Quentin-Fallavier, France).

### 2.10. Preparation of Palmitate Solution

Palmitate (Sigma, Saint-Quentin-Fallavier, France) was first coupled to bovine serum albumin (BSA; Saint-Quentin-Fallavier, France) and then fully dissolved in pure ethanol at 70 °C for a final concentration of 500 mmol/L. This stock solution was mixed in a pre-warmed BSA solution (10% *w*/*w*, 37 °C) to reach a final concentration of 5 mmol/L. The mixture clarification was obtained by incubating at 55 °C for 15 min twice. The final palmitate:BSA molar ratio was set at 3.2:1. The control vehicle was prepared in the same condition. A 10% *w*/*w*, BSA solution was added to an equivalent volume of ethanol to match with the final palmitate solution. The final concentration of ethanol was <0.05% by volume in all experiments.

### 2.11. Cell Viability

The cell viability was investigated on an XTT-based method (Cell Proliferation Kit II, Sigma-Aldrich, Saint-Quentin-Fallavier, France). Experimental procedures were set according to the supplier’s recommendations. The optical density was measured at 450 nm.

### 2.12. Red Oil Staining

Oil Red O solution (0.5% in isopropanol) was obtained from Sigma (Saint-Quentin-Fallavier, France) and staining was performed according to the supplier’s recommendations. Oil Red O solution was mixed with distilled water in a 3:2 ratio to obtain the working solution (0.2% in 60% isopropanol). Prior to staining, the cells were washed with PBS twice and fixed with 4% paraformaldehyde (30 min at room temperature). PFA solution was then discarded. The cells were washed twice more with water prior to incubation with isopropanol (60%) for 5 min and then with the working Oil Red O solution for 20 min. After 5 more washes with water, the cells were observed under the microscope.

### 2.13. Triglycerides Levels

Triglyceride content was determined in HepG2 using a triglyceride assay kit (Abcam, Paris, France—ab65336) according to the manufacturer protocol. The triglycerides were converted to free fatty acids and glycerol. The glycerol is then oxidized to generate a colorimetric (570 nm) product.

### 2.14. Cholesterol Levels in HepG2 Cells

Evaluation was performed in cell lysates using a cholesterol quantification kit (Sigma, Saint-Quentin-Fallavier, France—MAK043) according to the manufacturer recommendations. The total cholesterol concentration was assayed by a coupled enzyme reaction resulting in a colorimetric (570 nm)/fluorometric (λ_ex_ = 535 nm/λ_em_ = 587 nm) product related to the cholesterol content.

### 2.15. RNA Sequencing

RNA isolation: RNA from HepG2 cells was isolated using TRIZOL according to the supplier’s recommendations. RNA quality control: RNA purity and concentration were determined using a NanoDrop ND-2000 Spectrophotometer (Thermo Fisher Scientific) and Qubit fluorometer (Thermo Scientific, Villebon-sur-Yvette, France), respectively. The integrity of RNA was assessed on an RNA 6000 NanoChip using a 2100 Bioanalyzer (Agilent Technologies). RNA-seq Library preparation and sequencing: Poly-A selection was performed from 500 ng of total RNA with the NEBNext^®^ Poly(A) mRNA Magnetic Isolation Module (New England Biolabs, Evry, France); then, libraries were prepared using a NEBNext Ultra II Directional RNA Library Prep Kit (New England Biolabs) according to the manufacturer’s instructions. The library was amplified over 9 cycles and purified using Ampure XP beads (Beckman Coulter, Villepinte, France). Library concentrations were measured using a Qubit 2.0 Fluorometer in combination with a Qubit dsDNA HS Assay Kit (Thermo Fisher Scientific). The library quality was confirmed by size analysis on a Bioanalyzer 2100 instrument with DNA 1000 Assay reagent kit (Agilent Technologies). This size and quantity information was used for pooling the libraries in equimolar concentrations to normalize each library. Single-end, 75 cycle sequencing was performed using a NextSeq 500/550 High Output v2 kit (75 cycles) (Illumina, Evry, France). The FASTQ files were generated using bcl2fastq software (Illumina). RNA-seq data analysis: Reads were trimmed using Trimmomatic software (version 0.36) to filter poor quality reads and to cut poor quality bases and adapters. Filtered reads were then mapped to the reference genome (*Homo sapiens*, assembly GRCh38, release 107) using STAR (version 2.7.10). The STAR software was also used to produce the count matrix, assigning reads to features using transcriptome annotation from GTF file. Quality control statistics were summarized using MultiQC software. Descriptive analysis was performed with DESeq2 R package. Differential analysis was performed with edgeR R package. TMM normalization was applied. Functions glmQLFit and glmQLFTest were used to identify differentially expressed genes between the two groups (To and TP). Genes of interest were selected according to the significance (Benjamini–Hochberg correction, corrected *p*-value less than 0.05) and the effect size (fold-change). Gene lists were annotated using the WebGestalt tool (http://www.webgestalt.org/ (accessed on 27 September 2022)).

### 2.16. Fatty Acid Synthase Protein Expression (ELISA)

In HepG2 cells, Fatty Acid Synthase levels were evaluated in the cell lysates using a human fatty acid synthase ELISA kit (Abcam, Paris, France—ab279412), following the manufacturer’s instructions.

### 2.17. HMG-CoA Reductase Activity

The activity was evaluated using a colorimetric kit from Sigma (Saint-Quentin-Fallavier, France—CS1090). The cell lysates were incubated with both NADPH and HMG-CoA Reductase specific substrate, HMG-CoA. The oxidation of NADPH by the catalytic subunit of HMG-CoA Reductase leads to a decrease in absorbance at 340 nm which was measured using an EL_X_808 IU spectrophotometer (BioTek Instruments, Paris, France).

### 2.18. Cell Lysis

The lysis buffer was obtained by mixing 50 mmol/L Tris pH 7.8, 150 mmol/L NaCl, 0.5% sodium deoxycholate, and 1% NP40. The cell lysates were stored at −80 °C until ready for analyses.

### 2.19. Protein Quantification

The protein contents were assayed using the BCA Protein Assay Kit (Millipore, Molsheim, France, 71285-M). The BCA protein assay is a biuret-based reaction. The reduction of Cu^2+^ to Cu^+^ in the presence of proteins in an alkaline environment is related to the protein concentration. The chromogenic reagent bicinchoninic acid chelates the reduced copper and turns into a purple complex that absorbs at 562 nm.

### 2.20. Statistics

Prism V.7.0, 8.0 and V.9.4.1 (GraphPad Software) were used to run statistic tests and draw figures. The following statistic plan was applied: a Shapiro–Wilk normality test was used to determine whether the data was consistent with a Gaussian distribution. If the data was not distributed according to the normal distribution, a Kruskal–Wallis nonparametric test was used followed by Dunn test for post hoc comparison. When normal distribution and equal variance was assumed, measures were subjected to one-way or two-way ANOVA with Tukey’s test for multiple comparisons. In the case of a measurement repeated over time, differences between groups and time points were tested using a repeated-measure two-way ANOVA followed by Sidak’s post hoc test for multiple comparisons. If a piece of data was missing making it impossible to run a repeated-measures 2-way ANOVA, a mixed-effects analysis was used instead. Values are presented as the means ± SEM unless specified otherwise. The differences were considered statistically significant at *p* < 0.05 with * for *p* < 0.05; ** for *p* < 0.01; *** for *p* < 0.001; and **** for *p* < 0.0001; ns for *p* > 0.05.

## 3. Results

### 3.1. Effect of Totum-070 Supplementation on Serum Lipids in Hamsters

**Figure 1:**
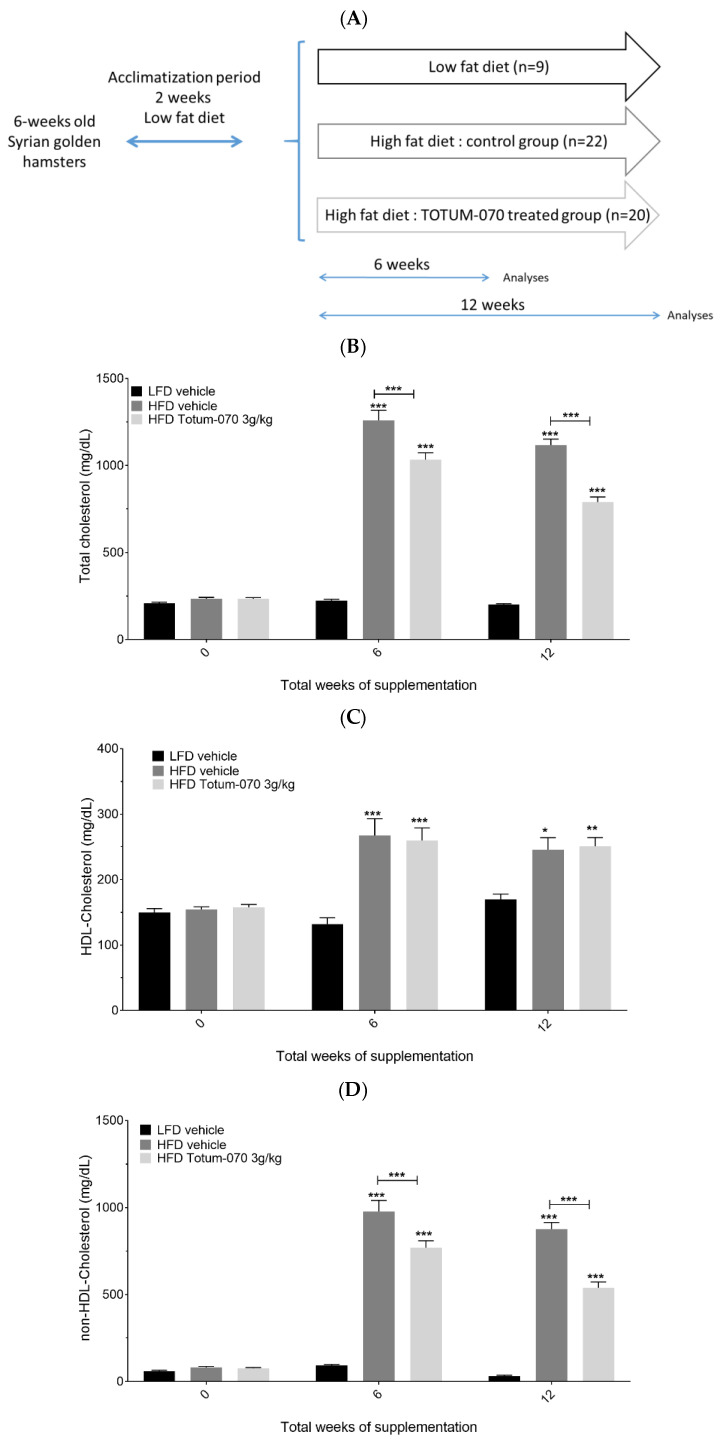

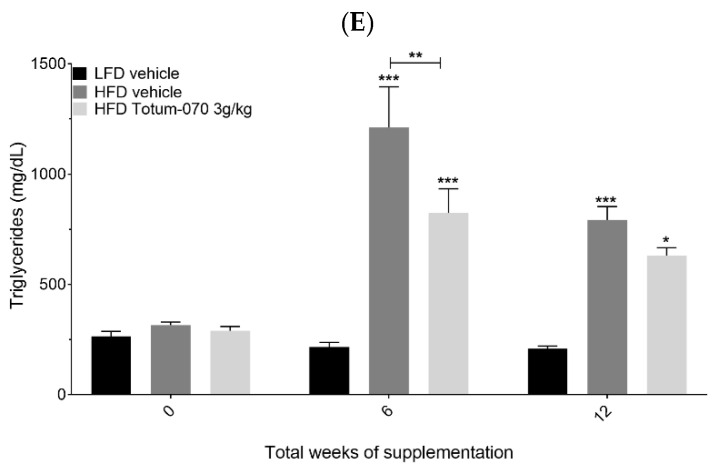
Effect of Totum-070 supplementation on circulating lipid parameters in high fat-fed hamsters. (**A**) Experimental design. (**B**) Total cholesterol. (**C**) non-HDL cholesterol. (**D**) HDL cholesterol. (**E**) Triglycerides. Values are presented as the means ± SEM unless specified otherwise. The differences were considered statistically significant at *p* < 0.05 with * for *p* < 0.05; ** for *p* < 0.01; *** for *p* < 0.001. Total cholesterol: global *p*-values (time < 0.001; column factor < 0.001; time × column factor < 0.001); F values (time F = 263; column factor F = 143; time x column factor F = 57.9); HDL cholesterol: global *p*-values (time < 0.001; column factor < 0.001; time × column factor = 0.01); F values (time F = 14.9; column factor F = 13.3; time × column factor F = 3.43); non-HDL cholesterol: global *p*-values (time < 0.001; column factor < 0.001; time × column factor = 0.01); F values (time F = 212; column factor F = 89.6; time × column factor F = 48.2); Total triglycerides: global *p*-values (time < 0.001; column factor < 0.001; time × column factor < 0.001); F values (time F = 17.2; column factor F = 18.4; time × column factor F = 5.40).

Eight-week-old Syrian golden hamsters were fed either HFD (45 kcal% fat, 1.4 gm% cholesterol) or control LFD (*n* = 9) for 12 weeks. Among the HFD-fed hamsters, some received 3 g/kg Totum-070 (*n* = 20) by gavage while others received vehicle (*n* = 22) (Figure 1A). There was no difference between the groups before the study (SW0) for all lipid parameters (Figure 1B–E). TOTUM-070 supplementation did not impact body weight nor energy intake (data are available as Supplementary Data, Figure S1A,B). As expected, HFD feeding induced robust hyperlipidemia at SW6 and SW12 compared to the lipid profile observed at SW0. Analysis of total cholesterol revealed a significant drop in the Totum-070 group compared with the HFD-vehicle group at SW6 (1259.47 ± 58.2 mg/dL HFD-vehicle vs. 1033.53 ± 39.3 mg/dL Totum-070, *p* < 0.001) and SW12 (1117.5 ± 34.3 mg/dL HFD-vehicle vs. 789.67 ± 29.8 mg/dL Totum-070, *p* < 0.001) (Figure 1B). Similar effects were observed in the non-HDL-cholesterol group, with a 21% decrease at SW6 (976.52 ± 64.4 mg/dL HFD-vehicle vs. 769.32 ± 39.7 mg/dl Totum-070, *p* < 0.001) and a 38.4% decrease at SW12 (875.67 ± 37.6 mg/dL HFD-vehicle vs. 539 ± 33.5 mg/dL Totum-070, *p* < 0.001) upon Totum-070 supplementation (Figure 1C). Given that Totum-070 did not alter HDL-Cholesterol levels (Figure 1D), these results indicated that the reduction in serum total cholesterol was driven by the effect of Totum-070 specifically on the non-HDL-cholesterol fraction. Triglyceride concentration was also measured (Figure 1E). In comparison with HFD-vehicle, Totum-070 reduced triglycerides by 32% at SW6 (1212 ± 183.5 mg/dL vehicle vs. 824.5 ± 109.1 mg/dL Totum-070, *p* < 0.01) and by 20.3% at SW12 (792.32 ± 61.2 mg/dL vehicle vs. 631.5 ± 34.L mg/dL Totum-070, ns), respectively. Altogether, the data demonstrated that Totum-070 prevented development of dyslipidemia in HFD-fed hamsters.

### 3.2. Kinetic Profile of TOTUM-070 Absorption in Humans

The clinical study was composed of two phases. In the first phase, we characterized in the blood stream the kinetics of the apparition of circulating metabolites following TOTUM-070 ingestion in order to (1) confirm the human absorption of the ingredient, (2) characterize the human metabolites originating from TOTUM-070 ingestion, and (3) determine the absorption profile that is mandatory to engage the second phase. In the second phase, we collected sera, before ingestion (naïve fraction) and at Tmax (enriched fraction), respectively. Both fractions were subjected to ex vivo investigations to evaluate the influence of human metabolites resulting from the consumption of TOTUM-070 on lipid metabolism in human hepatocytes.

**Figure 2:**
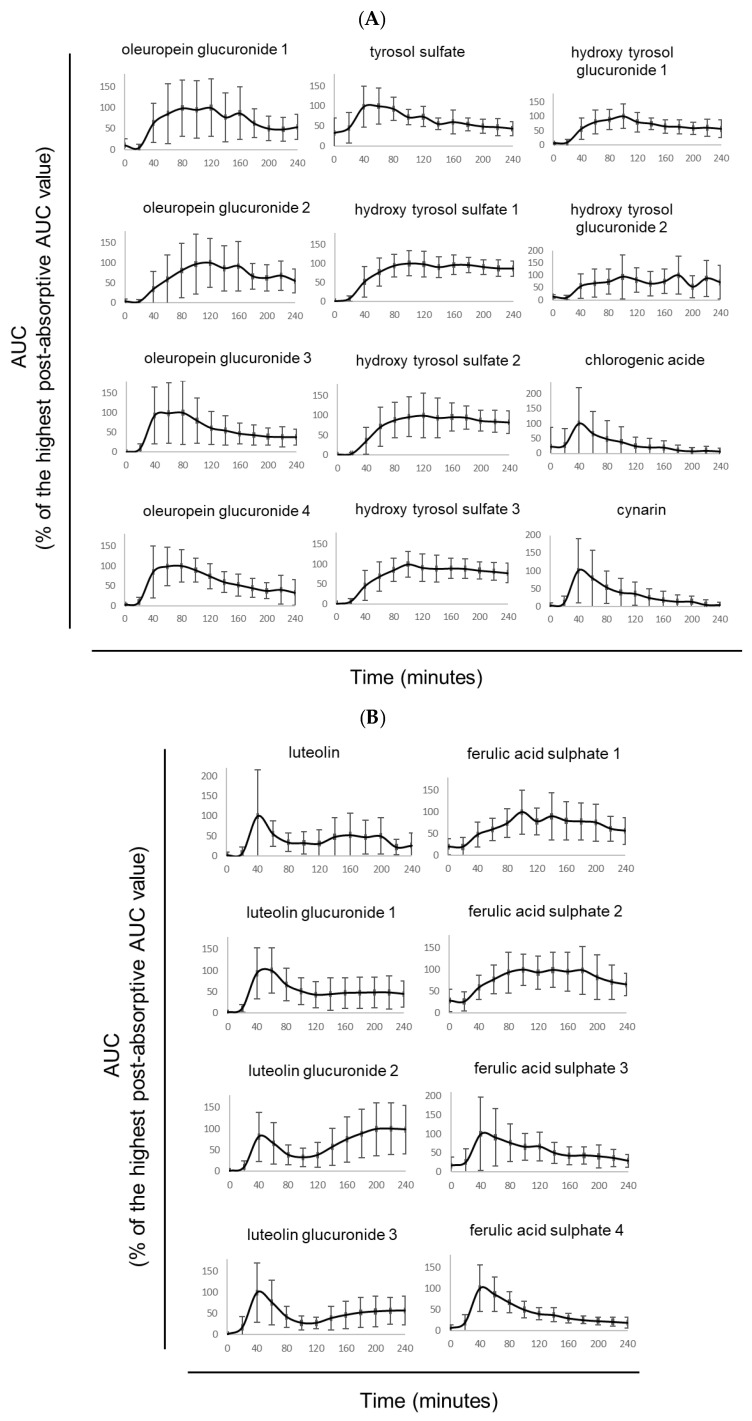

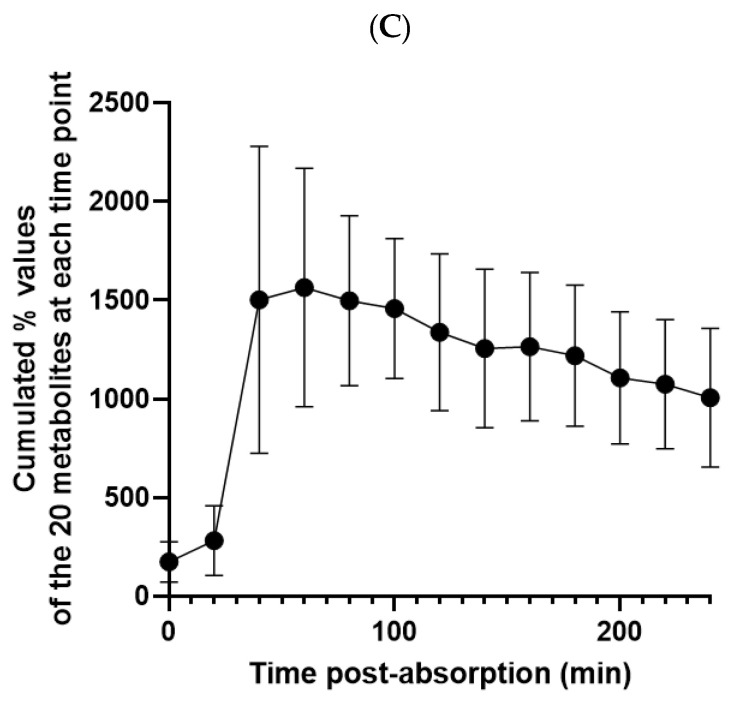
Absorption profile. (**A**,**B**) Circulating polyphenol metabolites resulting from human absorption and metabolization of TOTUM-070. For each metabolite, 100% is the highest post-absorptive AUC value. (**C**) Metabolites diversity per time point following TOTUM-070 ingestion (cumulated percentage values of the 20 metabolites at each time point ± SD).

The absorption and metabolization profile of the extract was monitored for 240 min. Modulation of the polyphenol metabolite concentrations in human serum was measured by UPLC-MS/MS (Figure 2A–C). We identified 20 detectable polyphenol metabolites. Figure 2A,B describe the kinetics of these metabolites found in human serum as a percentage of the highest area under curve (AUC) value observed. The metabolites include chlorogenic acid, cynarin, four ferulic acid sulfate isomers, luteolin, three luteolin glucuronides isomers, four oleuropein glucuronides isomers, tyrosol sulfate, three hydroxytyrosol sulfate isomers, and two hydroxytyrosol glucuronides isomers. The t-max (time to reach the maximum concentration observed in serum) ranged from 40 min to 120 min post-absorption depending on the type of metabolites (chlorogenic acid, cynarin, ferulic acids sulfate, luteolin, and luteolin glucuronides appearing faster than oleuropein glucuronides, tyrosol sulfate, hydroxytyrosol isomers, and hydroxytyrosol glucuronides isomers).

TOTUM-070 is a food ingredient composed of the combination of five plant extracts. This combination aims at uncovering synergistic effects of the derived metabolites. Therefore, to ensure a collection of serum fractions enriched with the highest diversity of metabolites, we plotted (Figure 2B) the cumulative percentages of each metabolite in order to represent metabolite diversity for each time point of the kinetics. According to Figure 2B, the cumulative percentages for all detected metabolites rapidly reached a peak between 40 and 60 min. To avoid serum collection during the raising slope, we chose 60 min rather than a 40 min time point. Consequently, enriched serum with TOTUM-070 metabolites was collected at 60 min post-ingestion for the second clinical phase.

### 3.3. Validation of the Ex Vivo Procedures

The final purpose of this clinical ex vivo investigation was to decipher whether human circulating metabolites following TOTUM-070 ingestion may impact human hepatocytes.

**Figure 3:**
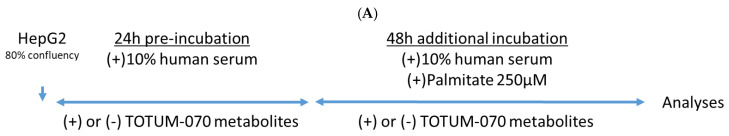

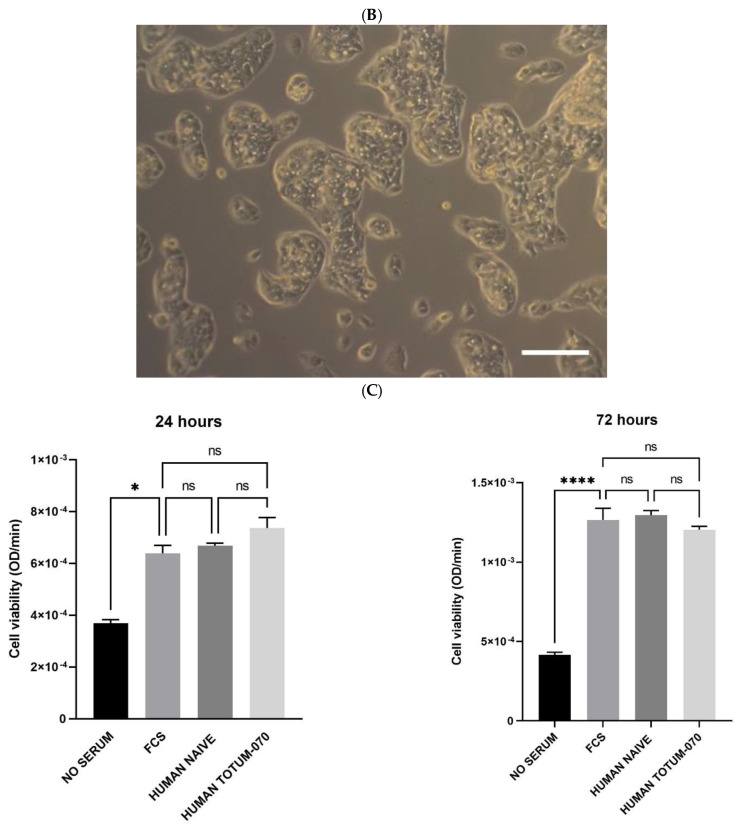
Human HepG2 hepatocytes subjected to ex vivo procedures. (**A**) Experimental design. (**B**) Phase contrast microscopy of human HepG2 hepatocytes. (**C**) Cell viability measured by XTT-based assay. Measures were performed in quadruplicates per condition/volunteer (*n* = 10 volunteers). Values are presented as the means ± SEM unless specified otherwise. The differences were considered statistically significant at *p* < 0.05 with * for *p* < 0.05; **** for *p* < 0.0001 and ns for *p* > 0.05. Cell viability 24 h (global *p*-value < 0.0001; F value = 10.44); 72 h (global *p*-value < 0.0001; F value = 69.82). Scale bar, 100 µm.

The cell culture protocols were designed as shown in Figure 3A. To ensure the biological soundness of this ex vivo approach, we verified cell growth and behavior in the presence of different human sera. In the presence of human serum, the cells took on a regular shape (Figure 3B). Regular fetal calf serum was used as a control reference. Cell growth consistently ceased in serum free cultures while it was maintained in the presence of FCS 10% (Figure 3C). Neither naïve nor enriched human serum exert any adverse effects on cells, and growth was similar to conventional fetal calf serum supporting further investigations.

### 3.4. Human Metabolites Deriving from TOTUM-070 Ingestion Limit Palmitate-Induced Intracellular Fat Deposit in Human Hepatocytes

Hepatocytes were subjected to 250 µM of palmitate to mimic a context of dyslipidemia and promote an alteration of cell lipid metabolism. This palmitate-related stress was performed after a preincubation of the cells either with naïve human serum or human serum containing circulating metabolites resulting from TOTUM-070 ingestion. The objective was to determine whether or not the presence of those circulating bioactives would be able to maintain or even improve lipid management by hepatocytes in such a lipotoxic environment.

**Figure 4:**
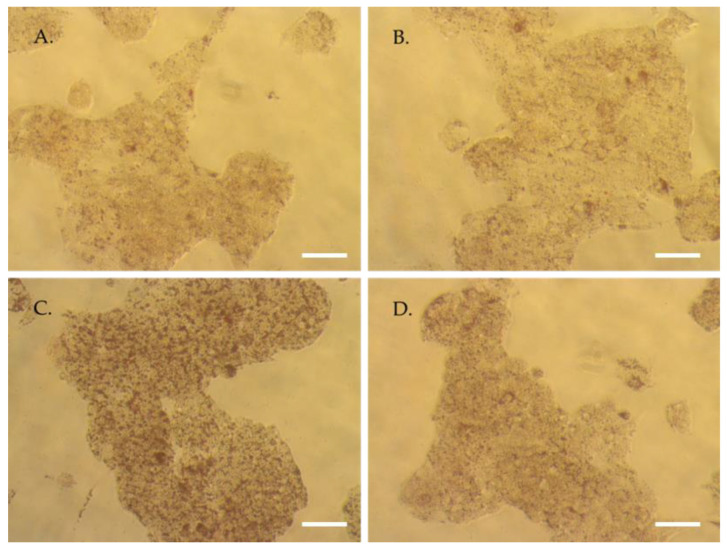
Human HepG2 hepatocytes Red Oil Staining. (**A**) Naïve human serum. (**B**) Human enriched serum with TOTUM-070 metabolites. (**C**) Naïve human serum + palmitate 250 µM. (**D**) Human enriched serum with TOTUM-070 metabolites + palmitate 250 µM. Scale bar, 100 µm.

We first checked the global lipid content of the hepatocytes. As witnessed by the red oil staining, baseline staining (Figure 4A) was not modified by the presence of the metabolites (Figure 4B). As expected, in the presence of palmitate, red staining was profoundly up-marked (Figure 4C) supporting an increase of intracellular lipid depots. Remarkably, in this lipotoxic context, the pre-incubation with human serum containing TOTUM-070 metabolites limited this augmentation almost back to baseline (Figure 4D). Quantifications are available as supplementary data (Figure S2).

### 3.5. Human Metabolites from TOTUM-070 Limits Triglycerides and Fully Abolish Palmitate-Related Hepatocytes Cholesterol Content

**Figure 5:**
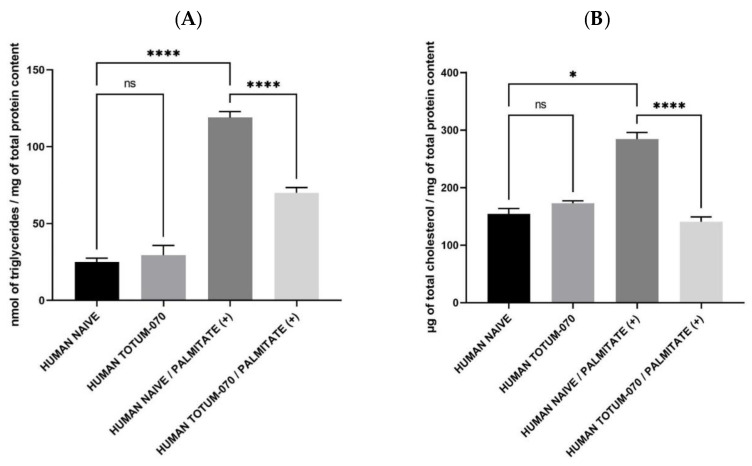
Quantification of intracellular lipid stocks. (**A**) Triglyceride content. (**B**) Cholesterol content. Measures were performed in quadruplicates per condition/volunteer (*n* = 10 volunteers). Values are presented as the means ± SEM unless specified otherwise. The differences were considered statistically significant at *p* < 0.05 with * for *p* < 0.05; **** for *p* < 0.0001 and ns for *p* > 0.05. Triglycerides: (global *p*-value < 0.0001; Kruskal–Wallis statistic = 59.31); Total cholesterol (global *p*-value < 0.0001; Kruskal–Wallis statistic = 52.95).

Based on this global lipid staining, we aimed at characterizing the type of lipid involved in these observations. Therefore, hepatocytes were subjected to the same experimental conditions (preincubation for 24 h either with naïve human serum or human serum containing circulating metabolites and an additional 48 h incubation with palmitate 250 µM in combination either with naïve human serum or human serum containing circulating metabolites) and harvested for intracellular triglyceride content. According to Figure 5A, the presence of TOTUM-070 metabolites did not influence intracellular TG content keeping it similar to the control baseline (in absence of palmitate). Palmitate induced a marked rise of TG content (+376%). While having no influence on control conditions, in this palmitate context, the presence of the circulating human metabolites deriving from TOTUM-070 ingestion efficiently limited the stock of intracellular triglycerides (+185%); in other words, the presence of metabolites was reduced by half the palmitate-induced rise of TG (Figure 5A).

Cholesterol metabolism by hepatocytes is a stepping stone in the management of hypercholesterolemia. Then, according to the aforementioned data in both rodents and ex vivo investigations on triglycerides, we checked the influence of TOTUM-070 human metabolites on cholesterol contents in hepatocytes. Similar to TG, TOTUM-070 metabolites had no significant impact on the cholesterol content baseline in the absence of palmitate. The presence of palmitate potently increased intracellular cholesterol levels (+84%). Here, in contrast to TG observations, the presence of the metabolites not only significantly limited but fully abolished the effects of palmitate pushing cholesterol content back to baseline.

### 3.6. TOTUM-070 Human Metabolites Greatly Impact the Expression of Genes Involded in Lipid Metabolism in Human Hepatocytes

Concentrating further on using the RNAseq approach, we investigated hepatocytes transcriptomic activity upon TOTUM-070 metabolites incubation. To allow accurate sequencing and analyses, we first checked the RNA integrity (data are available as supplementary data, Figure S3).

**Figure 6:**
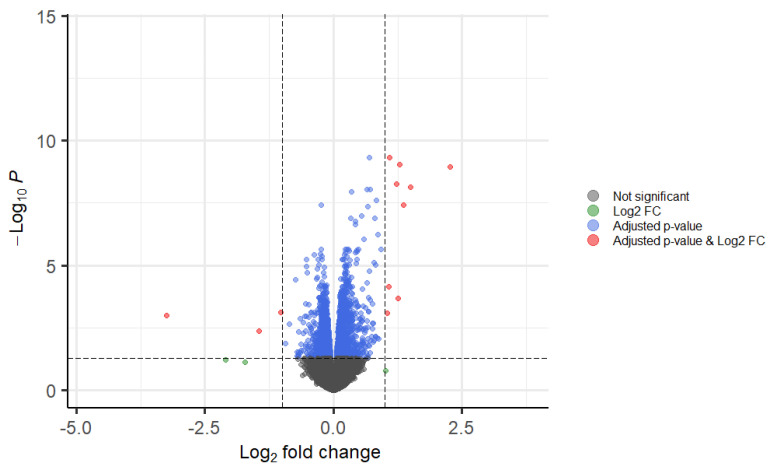
Volcano plot from RNA sequencing analysis. Genes with significant modulation are visualized with a volcano plot, to show statistical significance (adjusted *p*-values) versus magnitude of change (fold-change). Genes in red have a corrected *p*-value less than 0.05 and a log fold-change greater than 1.

According to the volcano plot, TOTUM-070 human metabolites greatly impacted hepatocyte transcriptomic activity (Figure 6). Genes were found to be differentially expressed when a corrected *p*-value less than 0.05 was obtained (with a Benjamini–Hochberg correction). A total of 1281 genes were found to be significantly repressed; 1603 genes were significantly enhanced; and 11,998 genes had their expression unchanged. Consistent with the impact of TOTUM-070 previously observed on intracellular lipid content, genes involved in fatty acid, cholesterol, or steroid metabolism were found among the top regulated targets with the highest enrichment ratio within gene clustering (Table 1). Since we could not show all the regulated genes, we have decided to join the whole list in the supplementary material (MS Excel file “Diff. Enriched_vs_Naïve”) that allows the reader to search for a specific target.

### 3.7. TOTUM-070 Derived Metabolites Limit Lipid Synthesis

Consistent with RNAseq data and in order to better understand how TOTUM-070 may exert its positive impact on lipid metabolism in hepatocytes, we checked for key enzymatic activity and expression involved in TG and cholesterol synthesis.

**Figure 7:**
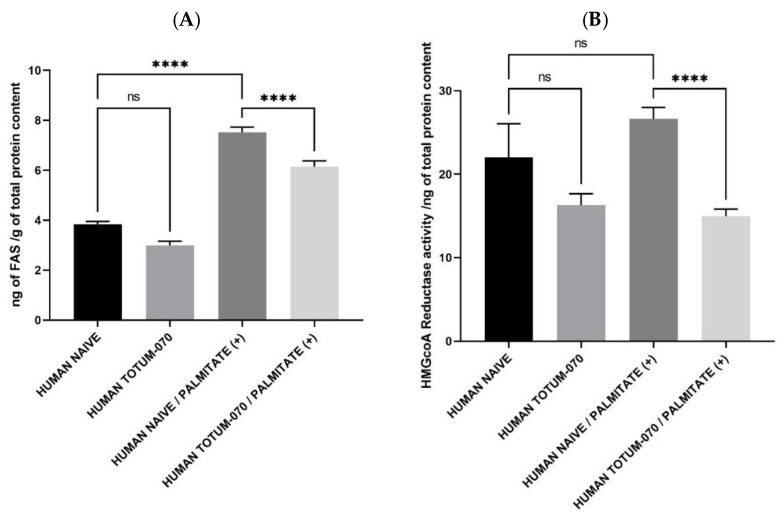
TOTUM-070 derived metabolites limit lipid synthesis. (**A**) Fatty acid synthase protein expression. (**B**) HMG coA Reductase activity. Measures were performed in quadruplicates per condition/volunteer (*n* = 10 volunteers). Values are presented as the means ± SEM unless specified otherwise. The differences were considered statistically significant at *p* < 0.05 with **** for *p* < 0.0001 and ns for *p* > 0.05. FAS: (global *p*-value < 0.0001; F value = 23.09); HMGCoA (global *p*-value < 0.0001; Kruskal–Wallis statistic = 35.25).

Amongst the protagonists of interest, fatty acid synthase expression was found to be down-regulated following RNAseq analysis (see supplementary material MS Excel file “Diff. Enriched_vs_Naïve”). Further investigation of protein expression confirmed this observation and paralleled nicely with the decrease of intracellular hepatocyte TG content (Figure 7A). Palmitate promoted FAS expression (+96%) in the presence of naïve human serum. This promotion was tempered when cells were cultured with human serum enriched with circulating TOTUM-070 metabolites (only +60% metabolites enriched human serum/human naïve serum control baseline). Thus, the presence of metabolites limited by one third the palmitate-induced up-regulation of the FAS.

HMG coA Reductase drives the de novo cholesterol synthesis in hepatocytes. In the absence of a lipotoxic context, TOTUM-070 metabolites exerted a slight but non-significant down-regulation on HMGcoARed activity compared to the control baseline (Figure 7B). In the absence of metabolites, palmitate promoted HMGcoARed activity without reaching a significant threshold. In contrast, in the presence of palmitate, metabolites significantly reduced HMGcoARed activity compared to naïve serum condition (−44%) and further support a TOTUM-070 related reduction of cholesterol level in a lipid-stressed environment. RNAseq analysis consistently showed that HMG coA Reductase (HMGCR) expression was down-regulated in the presence of TOTUM-070 metabolites (see supplementary material MS Excel file “Diff. Enriched_vs_Naïve”).

Taken altogether, these data support the observation that human metabolites derived from TOTUM-070 act to promote consistent enzymatic activities in hepatocytes in order to improve lipid management. In this light, they likely contribute to explain the inhibition of palmitate-induced increase of lipid content in human hepatocytes.

## 4. Discussion

In this manuscript, we first demonstrated, in a preclinical model, the potential health benefit of a plant blended extract on cholesterol metabolism within the context of dyslipidemia. Based on these preliminary data, we then aimed at investigating in humans the cellular basis supporting such a benefit, and we set up an ex vivo clinical trial that considers the full digestive process of nutrients [14,15,16,17,18]. We demonstrated, in a lipotoxic context that mimicked dyslipidemia and promoted an alteration of cell lipid metabolism, that human metabolites derived from TOTUM-070 were able to regulate transcriptomic and enzymatic activities and reduce TG and cholesterol intracellular content in human hepatocytes.

TOTUM-070 is a unique and patented blend of five different plant extracts including artichoke leaves, chrysanthellum, goji, olive leaves, and black pepper, all showing, separately, a latent effect on lipid metabolism. In order to further discuss the observed health benefit and biological effects, each plant extract composing TOTUM-070 will be compared with related extracts described in the literature. A traditional use of artichoke leaf extracts is reported, in particular for its antioxidant [22], choleretic, hepatoprotective [16], and hypolipidemic effects [23]. Some clinical studies [24,25,26] as well as the Cochrane review [27] have demonstrated a decrease in blood lipid concentrations after consumption of artichoke leaf extracts. In preclinical investigations, the consumption of an artichoke leaf extract by C57BL/6 mice (500 mg/kg of bodyweight) or Wistar rats (200–400 mg/kg of bodyweight) was reported to limit weight gain, adipocyte hypertrophy, or high levels of circulating triglycerides and cholesterol within a high fat diet [28,29]. Chrysanthellum (*Chrysanthellum indicum* subsp. *afroamericanum* B.L. Turner) reduces cholelithiasis and acts as a hypolipidemic agent [30]. The meta-analysis by Guo et al. (2017), examining the effect of *L. barbarum* (goji berries) on cardio-metabolic risk factors, concluded that *L. barbarum* significantly reduces total cholesterol and total triglyceride concentrations in the elderly population [31]. Interestingly, Goji composition exerts a low carbohydrate profile (only 20%) which may account for its role in reducing circulating triglycerides and total cholesterol while increasing HDL concentration [32,33]. Olive leaves extracts (*Olea europaea* L.) are known for preventing inflammatory chronic diseases due to the high content of various antioxidant molecules, such as oleuropein, flavonoids, and other phenolic compounds. Olmez et al. described a hypolipidemic effect in rats supplemented with olive leaf extract (50 or 100 mg/kg/day) for 8 weeks. LDLc and total cholesterol were significantly lower than when the rats were fed a high cholesterol diet [34]. In a randomized, double-blind, controlled crossover trial by Lockyer et al., olive leaf extract (providing 136.2 mg oleuropein and 6.4 mg hydroxytyrosol daily) significantly reduced plasma LDL cholesterol, total cholesterol, and triglycerides in 60 prehypertensive men after 6 weeks [35].

In this study, volunteers received a total of 4995 mg of the different plant extracts, and one may question the nutritional dimension of the dose used. For Goji, Cai’s 2015 study uses 300 mg/day of goji polysaccharides for 3 months [36]. The polysaccharide content for a goji dry extract is around 5 to 8% [37]. Thus, Cai’s dose corresponds to 3750 mg of dried fruit extract when the dose used for this project is only 552 mg. The single exposure to piperine from black pepper used in our study is 3 mg. It is much lower than the doses administered in the recent clinical studies of Heidari (7.5 mg/day over 4 weeks) [38] or that of Cicero (16 mg/day for 8 weeks) [39]. In Lockyer’s 2017 study, the dose of oleuropein given daily to volunteers for 6 weeks was 136 mg/d [35]. For our study, the dose used for the single dose was only 250 mg. Huber’s study shows the benefits of supplementing with a daily dose of 3.2 g of artichoke leaf extract given over 8 weeks [40]. For our study, the dose used for the single dose was only 2.24 g. Overall, the total quantity of polyphenols provided by 4995 mg of TOTUM-070 is estimated around 0.612 g. Since the recommended daily dose of polyphenols is about 1 g [21,41], the dose used in this study was quite relevant from a nutritional point of view.

To be health efficient while remaining nutritional, we hypothesized synergistic effects from the original blended composition of TOTUM-070. The relevance of nutritional synergies has already been reported in the literature. For instance, artichoke can potentiate the biological activity of bergamot phytosomes in mild hypercholesterolemia in the clinic [42]. ω-3 polyunsaturated fatty acids (PUFAs), ω-9 monounsaturated fatty acids (oleic acid), and phenolic compounds from olive cross-talk and act in synergy to contribute to the undebated Mediterranean diet’s cardiovascular benefits [43]. Moreover, we used black pepper (*Piper nigrum* L.) as part of the blend. It is a commonly consumed spice, but its active ingredient, piperine, also potentiates the bioavailability and bioefficacy of the product it is combined with [44].

In this study, the ingestion of TOTUM-070 led to the detection of about twenty circulating human metabolites including chlorogenic acid, cynarin, four ferulic acid sulfate isomers, luteolin, three luteolin glucuronides isomers, four oleuropein glucuronides isomers, tyrosol sulfate, three hydroxytyrosol sulfate isomers, and two hydroxytyrosol glucuronides isomers. Thus, these data give compelling evidence for efficient absorption and bioavailability of the product.

As mentioned earlier, some of these molecules were previously reported for improving lipid metabolism both in animals and humans; therefore, these circulating human metabolites likely contribute to the impact of TOTUM-070 on lipid management in human hepatocytes. Additionally, gut microbiota are important for the de-conjugation and excretion of biliary acids. In this light, puerarin, resveratrol, and quercetin are promising candidates for cholesterol elimination [45]. Interestingly, artichoke leaves [46,47] and goji berries [48] have been reported for their prebiotic properties exerting bifidigenic and anti-inflammatory capabilities, respectively. Although not explored in this study, such prebiotic attributes may play a role in the observed benefits on lipid and cholesterol metabolism.

Going back to liver function, RNAseq data reveal a massive impact of TOTUM-070 metabolites on hepatocyte transcriptomic activity. A total of 261 genes involved in different metabolic pathways were found to be modulated by the presence of TOTUM-070 circulating metabolites. The top regulated genes belong to clusters dealing with fatty acid and cholesterol metabolism, thus supporting both the potential health interest of the blend and the relevance of the pathophysiological target. In this light, our data support that human metabolites derived from TOTUM-070 act to promote consistent enzymatic activities in hepatocytes in order to limit lipid synthesis thus probably contributing to the inhibition of palmitate-induced increase of both triglycerides and cholesterol contents in human hepatocytes.

HMG coA Reductase drives the de novo cholesterol synthesis in hepatocytes. This endoplasmic reticulum (ER) membrane protein 3-hydroxy-3-methylglutaryl coenzyme A (HMG CoA) reductase produces mevalonate, the key intermediate in the de novo synthesis of cholesterol [49]. For this reason, HMG coA Reductase has been a major target for drug development. For instance, statins reduce cholesterol biosynthesis in the liver by inhibiting HMG-CoA reductase. Thus, statins have antiatherosclerotic effects that positively correlate with the percent decrease in LDL cholesterol [50]. Interestingly, luteolin that occurs naturally in *Olea europaea* leaves was also demonstrated to dock against HMG-CoA reductase and to reduce total cholesterol and LDL levels in a mice model of hypercholesterolemia [51]. According to metabolomic exploration, we detected both luteolin and three luteolin glucuronide isomers circulating after TOTUM-070 ingestion that may account for the observed effects on the limitation of HMG coA Reductase activity.

In order to support RNAseq analysis, we checked for a few key targets by RT-PCR, including CYP7A1 and LDLR. Neither RNAseq nor RT-PCR showed any significant level of regulation. Still, to further explore the potential benefit of TOTUM-070 metabolites on atherosclerosis onset, we checked the lecithin cholesterol acyltransferase (LCAT) activity that participates in the reverse cholesterol transport (RCT) mechanism: an “antiatherogenic” process [52] and an entry point for cholesterol elimination. The presence of circulating metabolites was found to consistently promote LCAT activity (data are available as Supplementary Data, Figure S4).

All together, these data provide valuable biochemical insights into the mechanisms underlying, how, in humans, metabolites deriving from TOTUM-070 ingestion may impact lipid management in liver cells in order to support preventive strategies in hypercholesterolemia and subsequent atherosclerotic onset. Further clinical investigations are now required to validate such a benefit upon chronic exposure with the ingredient TOTUM-070.

## 5. Conclusions

Using an innovative and acknowledged clinical ex vivo trial that fully considers the digestive processes of nutrients in humans, we further support the relevant health benefit of targeting nutrient synergies, and we provide clues on the role of circulating human metabolites produced following TOTUM-070 intake on lipid metabolism in human hepatocyte.

## 6. Patents

The human ex vivo methodology used in this study has been registered as a written invention disclosure by the French National Institute for Agronomic, Food and Environment Research (INRAE) (DIRV#18-0058). Clinic’n’Cell^®^ has been registered as a mark [14,15,16,17,18].

## Figures and Tables

**Table 1:** Enrichment ratio within gene clustering.

geneSet	Description	Overlap	enrichmentRatio	*p*-Value
hsa03010	Ribosome	87	3.8442376877825852	0
hsa03050	Proteasome	30	3.947339847991314	1.2612133559741778 × 10^13^
hsa00100	**Steroid biosynthesis**	10	3.1163209326247214	3.852518584954723 × 10^4^
hsa00220	Arginine biosynthesis	10	2.8195284628509385	0.0010621371529087043
hsa01040	**Biosynthesis of unsaturated fatty acids**	12	2.6315598986608757	7.355634194528005 × 10^4^
hsa01212	**Fatty acid metabolism**	21	2.5904417752442996	1.1116706622127381 × 10^5^
hsa00062	**Fatty acid elongation**	12	2.3684039087947886	0.002233923233486923
hsa04115	p53 signaling pathway	28	2.3026149113282663	6.596143085291217 × 10^6^
hsa04979	**Cholesterol metabolism**	18	2.131563517915309	8.662155586620646 × 10^4^
hsa00190	Oxidative phosphorylation	47	2.0923869119051703	1.684037256310944 × 10^7^
hsa03320	PPAR signaling pathway	26	2.080354784752179	1.0736921306331304 × 10^4^
hsa04714	Thermogenesis	78	2.0167631537772217	1.070485922127773 × 10^10^
hsa03040	Spliceosome	45	2.0033491709730353	1.2521413876864784 × 10^6^
hsa05012	Parkinson disease	47	1.9597708400238565	1.4871034260677263 × 10^6^
hsa05130	Pathogenic Escherichia coli infection	18	1.937785016286645	0.0029800691866965767
hsa05016	Huntington disease	62	1.9020860407419287	1.0991630317036538 × 10^7^
hsa04932	Non-alcoholic fatty liver disease (NAFLD)	47	1.8677010690160243	6.65595639137706 × 10^6^
hsa00240	Pyrimidine metabolism	31	1.8173396329860998	4.129936339646312 × 10^4^
hsa04211	Longevity regulating pathway	27	1.7962613915016652	0.0011583288792791357
hsa03008	Ribosome biogenesis in eukaryotes	24	1.7543732657739173	0.0030116440823730883
hsa05222	Small cell lung cancer	27	1.737687650474437	0.0019929850989139908
hsa05010	Alzheimer disease	49	1.6966636188734594	7.322855774782866 × 10^5^
hsa04152	AMPK signaling pathway	33	1.628277687296417	0.0022440329320105867
hsa04218	Cellular senescence	41	1.5172587540716613	0.0030390963377235902
hsa05169	Epstein-Barr virus infection	50	1.4728880029818336	0.002243922870621695
hsa01100	**Metabolic pathways**	261	1.184201954397394	6.418137152468528 × 10^4^

## Data Availability

The data presented in this study are available on request from the corresponding author. The data are not publicly available due to ethical restrictions.

## Supplementary Materials

The following are available online at https://www.mdpi.com/article/10.3390/nu15081903/s1, Table S1: UHPLC-MS/MS parameters for the detection of phenolic metabolites in serum; Figure S1: Caloric intake (A) and body weight (B) of hamsters upon high fat diet with or without TOTUM-070 supplementation; Figure S2: Red Oil staining quantification; Figure S3: RNA integrity check for sequencing; Figure S4: LCAT activity (enzymatic assay). Data source for RNAseq: excel file “Diff. Enriched_vs_Naïve”.
