# Supplementary material for: Circulating Human Metabolites Resulting from TOTUM-070 Absorption (a Plant-Based, Polyphenol-Rich Ingredient) Improve Lipid Metabolism in Human Hepatocytes: Lessons from an Original Ex Vivo Clinical Trial
Source: Nutrients. 2023 Apr 14;15(8):1903. doi: 10.3390/nu15081903 (PMC10145174; doi:10.3390/nu15081903)
Supplement: Supplementary file 1 [file nutrients-15-01903-s001.zip › nutrients-2310522-supplementary.pdf]

Table S1. UHPLC-MS/MS parameters for the detection of phenolic metabolites in serum

Metabolite	MRM transition	tR (min)
Oleuropein glucuronide-isomer01	553.0 -> 377.0	9.30
Oleuropein glucuronide-isomer02	553.0 -> 377.0	9.65
Oleuropein glucuronide-isomer03	553.0 -> 377.0	12.00
Oleuropein glucuronide-isomer04	553.0 -> 377.0	12.17
Tyrosol sulfate	217.0 -> 137.0	9.50
Hydroxytyrosol sulfate-isomer01	233.0 -> 153.0	2.60
Hydroxytyrosol sulfate-isomer02	233.0 -> 153.0	1.23
Hydroxytyrosol sulfate-isomer03	233.0 -> 153.0	4.60
Hydroxytyrosol glucuronide-isomer01	329.0 -> 153.0	2.60
Hydroxytyrosol glucuronide-isomer02	329.0 -> 153.0	7.50
Luteolin	285.0 -> 133.0	12.90
Luteolin glucuronide-isomer01	461.0 -> 285.0	9.66
Luteolin glucuronide-isomer02	461.0 -> 285.0	9.30
Luteolin glucuronide-isomer03	461.0 -> 285.0	9.95
Chlorogenic acid	353.0 -> 191.0	1.45
Cynarin	515.0 -> 353.0	8.21
Ferulic acid sulfate -isomer01	273.0 -> 193.0	9.30
Ferulic acid sulfate -isomer02	273.0 -> 193.0	9.60
Ferulic acid sulfate -isomer03	273.0 -> 193.0	7.82
Ferulic acid sulfate -isomer04	273.0 -> 193.0	7.90

Figure S1: Caloric intake (A) and body weight (B) of hamsters upon high fat diet with or without TOTUM-070 supplementation

A.

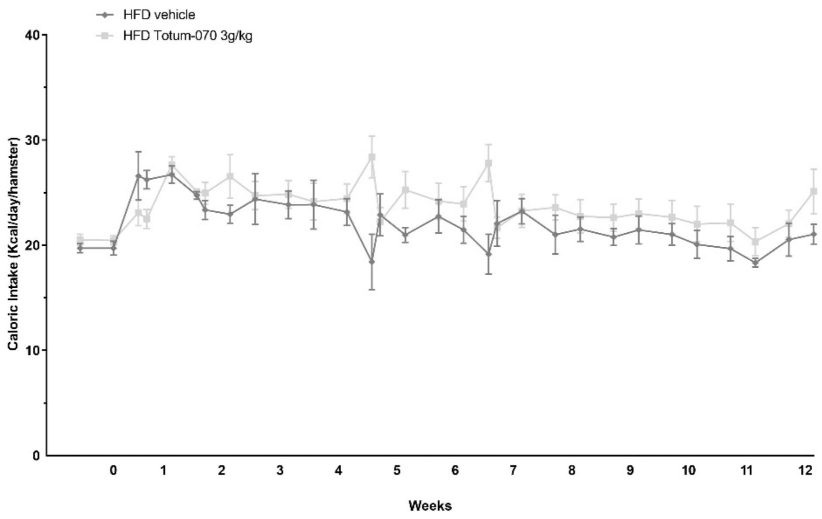

B.

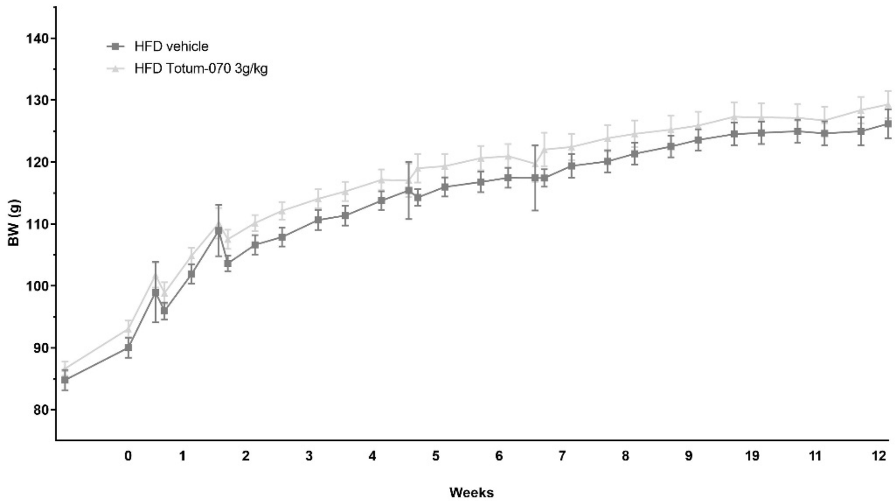

6-weeks old Syrian golden hamsters were received and housed at the Valbiotis animal facility (Valbiotis, Riom, France) under standard 12-h light and 12-h dark cycle. Upon arrival, all hamsters were fed a low-fat diet (LFD, D18102403, Research Diet, New Bruns-wick, NJ, USA) for 2 weeks of acclimatization until the age of 8 weeks. Thereafter, hamsters placed on a high fat high cholesterol diet (HFD: 45kcal% Fat mainly hy-drogenated coconut oil, 17kcal% Fructose, 1.4gm% cholesterol, D99122211, Research Diet, New Brunswick, NJ, USA) were divided in two groups: a control group (n=22) and a treated group supplemented with TOTUM-070 (n=20). TOTUM-070 solution was prepared in vehicle tap water Tween20 1% at 300 g/l. Administration of TOTUM-070 in HFD hamsters was performed by daily gavages at 3 g/kg body weight. The HFD control group was gavaged with vehicle only. Food and water were supplied ad libitum. Study duration was completed after 12 weeks of supplementation.

Figure S2: Red Oil staining quantification

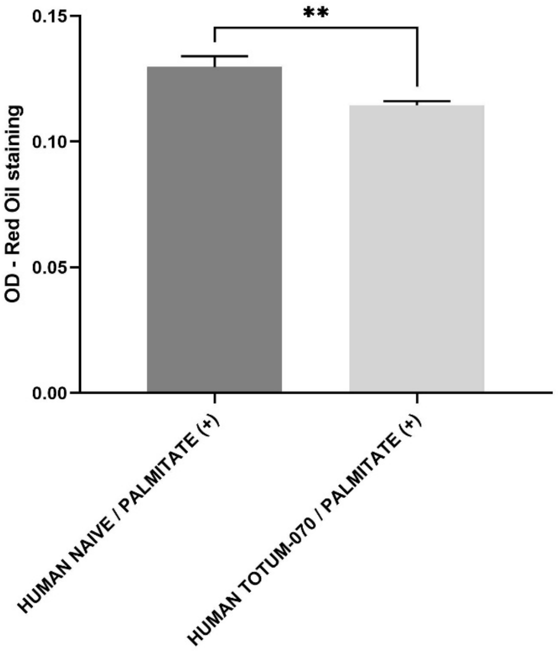

In order to quantify the staining, the fixed dye was redissolved in a fix volume of 100% isopropanol and optical density was measured at 490 nm on an ELX808 IU spectrophotometer (BioTek Instruments, USA). Prism V.9.4.1 (GraphPad Software) were used to run statistic tests and draw figures. The following statistic plan was applied: A Shapiro–Wilk normality test was used to determine whether the data are consistent with a Gaussian distribution. When data were not distributed according to the normal distribution, thus a nonparametric Wilcoxon paired t-test was used. When normal distribution and equal variance was assumed, measures were subjected to unpaired t-test. Values are presented as the means ± SEM. The differences were considered statistically significant at $p< 0.05$ with ** for $p< 0.01$. Two-tailed exact *p-value = 0.0039.*

Figure S3: RNA integrity check for sequencing

RNA quality is checked with Agilent 2100 Bioanalyzer which uses a combination of microfluidics, capillary electrophoresis, and fluorescent dye that binds to nucleic acid to evaluate both RNA concentration and integrity. The software assigns a specific quality number to RNA samples: RNA Integrity Number (RIN). Sample quality is good with RIN values ranging from 9.2 to 9.7.

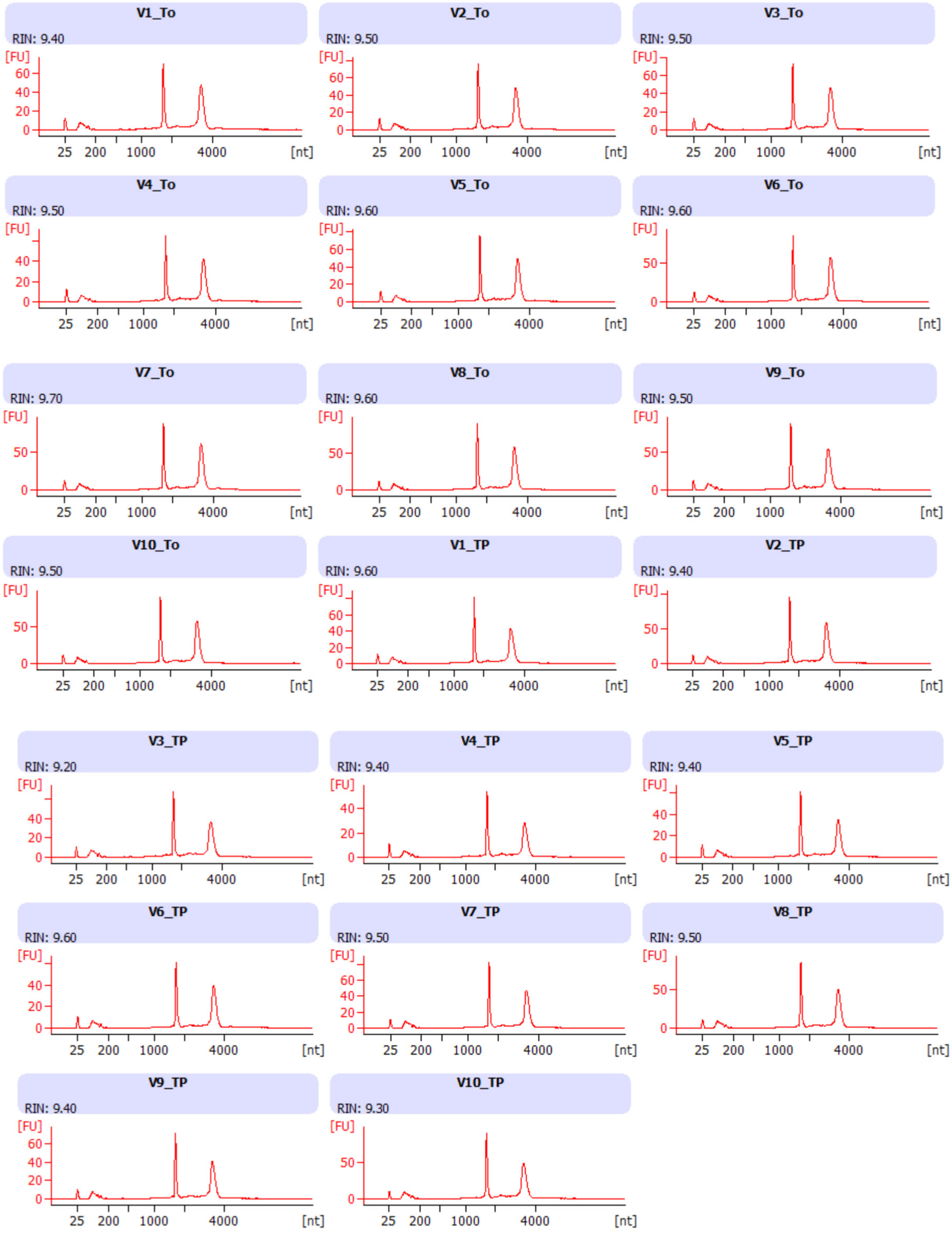

Figure S4: LCAT activity

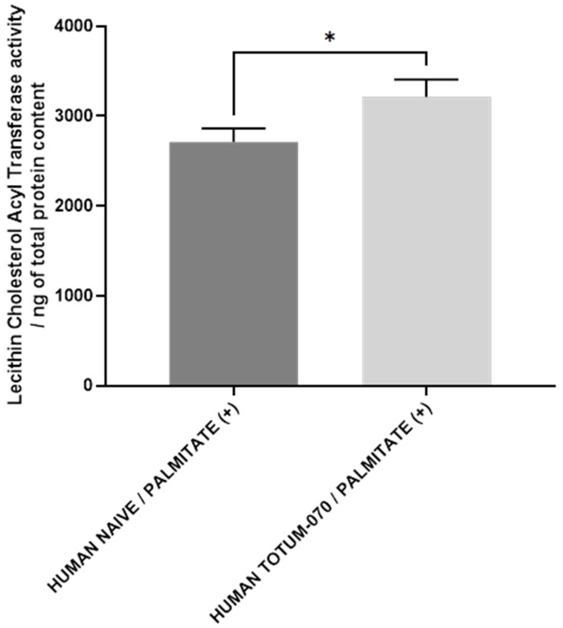

A. Lecithin Cholesterol AcylTransferase (LCAT) Activity was determined in HepG2 cell culture supernatant using a commercial kit from Sigma (MAK107) according to the manufacturer protocol. Briefly, supernatants were incubated for two hours and a half at 37°C with a fluorescent substrate (λex=360 nm / λem=460 nm). By altering the substrate, LCAT activity induces a loss of fluorescence which was evaluated in the samples.

Prism V.9.4.1 (GraphPad Software) were used to run statistic tests and draw figures. The following statistic plan was applied: A Shapiro–Wilk normality test was used to determine whether the data are consistent with a Gaussian distribution. When data were not distributed according to the normal distribution, thus a nonparametric Wilcoxon paired t-test was used. When normal distribution and equal variance was assumed, measures were subjected to paired t-test. Values are presented as the means ± SEM. The differences were considered statistically significant at $p < 0.05$ with * for $p < 0.05$. Two-tailed exact *p-value = 0.0422*.
